# A working model of stroke recovery from rehabilitation robotics practitioners

**DOI:** 10.1186/1743-0003-6-6

**Published:** 2009-02-25

**Authors:** Hermano Igo Krebs, Bruce Volpe, Neville Hogan

**Affiliations:** 1 Mechanical Engineering Department, Massachusetts Institute of Technology, Cambridge, MA, USA; 2 Department of Neurology and Neuroscience, Burke Institute of Medical Research, Weill Medical College, Cornell University, White Plains, NY, USA; 3 Department of Neurology, University of Maryland, School of Medicine, Baltimore, MD, USA; 4 Brain and Cognitive Sciences, Massachusetts Institute of Technology, Cambridge, MA, USA

## Abstract

We reviewed some of our initial insights about the process of upper-limb behavioral recovery following stroke. Evidence to date indicates that intensity, task specificity, active engagement, and focusing training on motor coordination are key factors enabling efficacious recovery. On modeling, experience with over 400 stroke patients has suggested a working model of recovery similar to implicit motor learning. Ultimately, we plan to apply these insights in the development of customized training paradigms to enhance recovery.

## Introduction

Rehabilitation robotics has begun to realize its promise that delivery of high-dosage, guided movement protocols will alter the impairment (neurological deficit) phase of modern post-stroke therapy. However, the mechanisms of this motor performance enhancement remain unclear. Recovery, which occurs spontaneously and continues imperfectly, depends on a myriad of biological and social/economic factors: age, gender, physical and mental health, size and location of lesion, family and other support networks, insurance, income, and probably many more. A quantitative, scientific understanding of the mechanisms of the post-stroke recovery process is the key to improving the speed and ultimate level of recovery. By design, robotics provides a reliable, controllable, objective instrument platform from which to deliver high intensity therapy and to characterize recovery at the behavioral level. By controlling the amount of therapy and quantitative characterization of recovery, it will allow us to determine the optimal therapy for a particular patient's needs. It will also enable a richer set of therapies complementary to existing ones and novel cellular, electrophysiological and pharmacological interventions.

We recently reviewed some initial insights about the process of upper-limb behavioral recovery following stroke that have emerged from our robotics work [1]. Evidence to date indicates that intensity and task specificity are key factors enabling efficacious recovery [2]. However, our results suggest that the dynamics and form of therapy – as well as its intensity (dosage) – are critical. We showed that robotic driven muscle strengthening is beneficial, but other forms of robotic training emulating concepts of motor-learning appear to lead to better outcomes in terms of movement coordination [1,3] and, that passive movement was insufficient to alter motor recovery, since high intensity passive movement therapy did not promote superior outcome over low intensity passive movement [4]. Hence, we conclude that patients must be actively engaged and attempting to move. Together these results suggest that focusing therapy on movement coordination rather than muscle strengthening may be the most appropriate general approach for robotic therapy and that sensorimotor therapy may operate by helping patients "relearn" motor control, reinforcing the widely-held belief (albeit usually implicit) that recovery is like motor learning. Indeed, motivated by the literature on motor learning [5] and the classical Hebbian notion that experience modulates synaptic strength, we developed and tested a novel patient-responsive protocol that progressively adapts robotic training and assistance (based on measures of movement coordination) to continually challenge patients without overtaxing them. That protocol yields substantially improved outcomes [6,7] beyond the previously reported benefits of repetitive robotic therapy.

At the same time, we recognize that simplistic ideas based on motor adaptation and learning may be insufficient to describe the complex process of recovery after neurological injury. The strongest evidence to support this statement is the ubiquitous incidence of abnormal muscle tone, spasticity, and abnormal synergies during recovery. It seems tritely obvious that unimpaired adults learning a new motor skill do not have to contend with these difficulties, yet they are so common in recovery after neurological injury as to be characteristic of the process. These abnormalities warrant study in their own right. Our work to date has shown that the conventional clinical perception of abnormal synergies – that they are exhibited early in recovery and must be suppressed or "broken" if recovery is to gain momentum – may not stand up to close quantitative scrutiny. Our analysis indicates that synergies are not first expressed and then "broken" during recovery; instead, they are present throughout but with a "gain" or magnitude that varies as recovery proceeds [8,9].

Nevertheless, we acknowledge the appeal of motor learning as the basis of a theory of motor recovery, though it may need careful refinement to serve as a basis for designing new therapies. Here, we will attempt to refine the idea of motor recovery as a process of motor re-learning and to present a "working model" (admittedly speculative) of the process of neuro-recovery. We will provide neither an overview of our different robots nor a discussion of the multitude of robotic devices designed elsewhere following our pioneering robotic module, MIT-Manus. Comparisons of alternative robotic design philosophies and summaries of past clinical results, including several meta-analyses, can be found elsewhere [10-14].

## Leaving the ivory tower

From the outset we recognized that the successful development of rehabilitation robotics required a multi-disciplinary effort. We had to abandon the "comfort zone" of our academic elitism at engineering laboratories and engage with clinicians and patients at rehabilitation facilities. We recognized that we had to abandon our Ivory Towers and establish well-balanced multidisciplinary collaborations. In fact, we perceive that the single greatest weakness of the plethora of different therapeutic robot designs that have emerged recently – some quite ingenious and technically appealing – is the lack of a truly balanced multi-disciplinary team to establish objectively verified and clinically meaningful target requirements. A similar (though perhaps more recent) weakness is evident in several attempts to apply mathematical modeling and computational neuroscience to describe recovery and prescribe treatment. For example, one very ingenious suggestion is to capitalize on the after-effects of adaptation to novel mechanical environments so as to induce beneficial changes in patients' motor behavior [15]. However, the practicality of this theoretical approach remains unclear. In the first place, even in unimpaired subjects, the duration of these after-effects is fleeting at best. Secondly, to the best of our knowledge, there is no clinical evidence to support this approach as a practical means of delivering therapy for patients with severe paresis. Other examples that might profit from wider collaboration include the idea of moving the "system" beyond local minima and encouraging the delivery of therapy only when patients reach a period of performance stagnation [16]. In effect, this untested bi-stable mathematical model proposes rationing therapy while patients are improving and, consequently, might limit the ultimate potential of recovery. One must realize that such a simplistic two-attractor model could not stand against actual clinical data. There is no substitute for hands-on experience and the opportunity to listen to experts, i.e., the patients.

## Listening to experts

Since 1994, we have had the privilege of spending significant time with over 400 stroke patients at multiple institutions. Patients would describe their crisp, clear understanding of the goals of training and the frustrations of being unable to execute them. They understood the "games" used for robotic therapy and their simple objectives, but seethed with frustration and anger at their inability to perform the appropriate movements to accomplish the goals. Unless the impact of stroke is resolved within the initial 24 hours, impairments linger. Yet they appear to be amenable to the acquisition of new skills without awareness of the learned information over repetitive trials. The insightful self-assessment of stroke by Brodal should be required reading for all researchers interested in stroke recovery [17]. Quoted here are some of his statements on skilled movements: "Under normal conditions the necessary numerous small delicate movements had followed each other in the proper sequence almost automatically, and the act of tying (as in a bow-tie) when first started had proceeded without much conscious attention. Subjectively the patient felt as if he had to stop because his fingers did not know the next move. He had the same feeling as when one recites a poem or sings a song and gets lost. The only way is to start from the beginning. It was felt as if the delay in the succession of movements (due to pareses and spasticity) interrupted a chain of more or less automatic movements. Consciously directing attention to the finger movements did not improve the performance; on the contrary it made it quite impossible."

We believe this expert's insight can be translated into working models of motor recovery. First of all, his description entices further research into models fractionating motor control and how this may be deranged by stroke [5,18-20] and also into models that implicate a sequence of movement units or submovements underlying functional motor performance. We have written about that possibility of submovement model elsewhere [21,22] and will not repeat the discussion in this manuscript. Secondly, this description strongly suggests that the process of neuro-recovery following stroke has some characteristics of *implicit motor learning*, in which subjects understand the goals but are unable to comprehend how to activate their muscles to achieve those goals. Our paper will focus on the latter.

## Implicit motor learning

If human learning can be divided into so-called declarative and procedural forms, then declarative or explicit learning and memory refer to the acquisition and retrieval of information accompanied by awareness of the learned information and its influence. Explicit learning is most often put into practice through language functions [23]. Procedural or implicit learning and memory refer to acquisition without awareness of the learned information and its influence [24,25]. Naturally there are structure-function correspondences that have been demonstrated for these dichotomous human behaviors [26]. We and others postulate that stroke motor recovery has similarities to implicit motor learning [27] and in particular, "procedural motor learning", a form of implicit learning where skill improves over repetitive motor trials. It is worth noticing that this definition is somehow imprecise as both implicit learning and adaptation could equally apply. We will exact the difference later.

In previous work, we reported on the integration of robotic technology with functional brain imaging to study whether the unskilled phase of procedural learning of a motor task (early learning) involves areas of the brain distinct from those involved in a more skilled learning phase of the task (late learning) in young healthy right-handed subjects [28,29]. PET was used to measure aspects of neural activity underlying learning of the motor task, while a portable robotic device was used to generate a "virtual mechanical environment" that subjects learned to manipulate. This drew upon an elegant line of study [30] using a robotic device originally developed in our laboratory [31] to generate a force field that responded to the subjects' arm movements, thereby generating a "haptic virtual environment" that subjects learned to manipulate.

We found during a right-handed task in young unimpaired subjects that early learning activated the right striatum and right parietal area, as well as the left parietal and primary sensory area, and that there was a deactivation of the left premotor area. As subjects became skilled at the motor task (late learning), the pattern of neural activity shifted to the cortico-cerebellar feedback loop, i.e., there was significant activation in the left premotor, left primary motor, and sensory areas, and in the right cerebellar cortex. These results support the notion of different stages of implicit motor learning (early and late implicit learning), occurring in an orderly fashion at different rates. Moreover, these findings indicate that the cortico-striatal loop plays a significant role during early implicit motor learning, whereas the cortico-cerebellar loop plays a significant role during late implicit motor learning [32]. These classes of motor learning behaviors have also been demonstrated in skill learning in unimpaired subjects, where a decidedly different fMRI activation pattern resulted after the subject experienced training and could depend on implicit motor information [33-37]. Of course, one must take with appropriate caveats the application of this stark model inter-playing the purported role of the cortico-striatal and cortico-cerebral loops on implicit motor learning to motor rehabilitation. We must take into consideration, when designing a flexible rehabilitation program, that there are many and significant co-morbid cognitive factors involved and these might limit recovery [38-40].

## Implicit motor learning as a model for neuro-rehabilitation following stroke

We have assessed the competence of this working model to account for clinical experience with patients recovering from stroke. Here we will present a few selected pieces of data that appear to support the model. Our procedural motor leaning experiments performed with PET metabolic and blood flow information revealed that the cortico-striatal loop played a significant role during early learning and motor plan transition, while the motor execution areas played a significant role during late motor learning (cortico-cerebellar). If motor recovery has similar traits to implicit motor learning, then we speculate that patients with basal ganglia lesions would take longer to start the recovery process (i.e., be deficient in the early recovery phase). Conversely, patients with lesions in the motor execution areas would recover more slowly during later phases (i.e., be deficient in the late recovery phase). Of those patients with lesions in the motor execution areas, the smaller the number of structures affected, the better the outcome expected in the late recovery phases.

These predictions do not speak to the ultimate potential of recovery but to the pattern of recovery. Intuitively one might expect that larger lesions would lead to slower recovery. However, Miyai and colleagues showed that, in fact, patients with smaller lesions confined to the basal ganglia (CS) have diminished response during the sub-acute rehabilitation period compared to patients with much larger lesions that involve cortical and subcortical territories (CS+) [41]. Miyai suggested that basal ganglia strokes might cause persistent corticothalamic-basal ganglia interactions that are dysfunctional and impede recovery, which is consistent with our prediction for the influence of these motor control brain regions during early recovery. But our predictions extend beyond the subacute phase. Our working model suggests that strokes confined to the basal ganglia should have minimal impact during the late recovery phase and not preempt recovery, while large strokes involving the motor execution areas should preempt late recovery.

For example, from our initial study delivering rehabilitation robotic therapy to 20 sub-acute patients, the comparison of outcome for 5 patients with corpus striatum lesions (CS) versus 6 patients with corpus striatum plus cortex (CS+) is shown in Table 1[42]. These patients had comparable demographics and were evaluated by the same therapist on hospital admission (19 days ± 2 post-stroke), discharge (33 days ± 3 later), and follow-up (1002 days ± 56 post discharge). As in Miyai et al's study, the CS group had smaller lesion size (CS = 13.3 ± 3.9 cm^3^, CS+ = 95.1 ± 25.2 cm^3^, p < 0.05). We found that early recovery shows a trend to progress at a slower pace for those with smaller lesions (CS) compared to those with larger lesions (CS+). While non-significant, our clear trend with this small sample size is inline with Miyai's very counter-intuitive result. To our knowledge, prior to his finding, the traditional wisdom had generally been that smaller lesion leads to better outcome. As we showed in Table 1, this conventional wisdom is actually correct in the long term, but not during the initial 12 weeks post-stroke.

**Table 1:** Change during Acute Rehabilitation & Follow-Up: Lesion Site Classification and Clinical Scales

Group	FMA (out of 66) Mean ± sem		MP (Out of 20) Mean ± sem		MS1 (Out of 40) Mean ± sem	
	Δ1	Δ2 *	Δ1	Δ2	Δ1	Δ2 *

CS(n = 5)	9.3 ± 5.4	25.0 ± 7.5	2.1 ± 1.2	6.1 ± 1.3	1.0 ± 3.3	16.0 ± 16.6

CS+(n = 6)	10.7 ± 2.8	-1.3 ± 2.4	4.3 ± 1.6	2.8 ± 2.2	7.7 ± 2.8	4.2 ± 1.8

Effect Size r	r = 0.15 small	r = 2.45 large	r = 0.60 large	r = 0.77 large	r = 0.94 large	r = 1.80 large

FMA – the Fugl-Meyer Assessment, MP the Medical Research Council Motor Power, MS1 the Motor Status Score for the shoulder and elbow. Δ1: score change from rehabilitation hospital admission to discharge; Δ2: score change from discharge to follow up; with p < 0.05 for statistical significance (*). Both parametric and nonparametric analyses were performed, and each yielded similar results. For conciseness, we have chosen to report our parametric analyses of the change scores here. Analyses of variance was used to compare changes during sub-acute phase (Δ1) and from hospital discharge to 3-years follow-up (Δ2) among the two lesion type groups. Nonparametric Mann-Whitney Tests compared changes in FMA, MP, and MSS scores for Δ1 and Δ2. StatView (SAS Institute, Inc., Version 5.0.1) was used for data analysis. The strength, or magnitude, of our findings was determined by calculating the effect size r. According to Cohen, r = .10 is a small treatment effect, r = .30 or greater represents a moderate effect, and r = .50 or greater is a large effect.

Our results are also consistent with our working model that during late recovery, lesions in the basal ganglia do not preempt improvement while the converse is true for lesions in the motor execution areas. Note in Table 1 that consistent with Miyai, the CS+ group appears to outperform the CS group during sub-acute rehabilitation (early recovery). However at follow-up, patients with smaller lesions fared statistically significant better. The CS group outperformed the CS+ group between discharge from the sub-acute hospital and follow-up (late recovery). Furthermore, consistent with our working model that motor execution areas are important during late recovery, the CS+ group improved little from discharge to follow-up.

**Figure 1:**
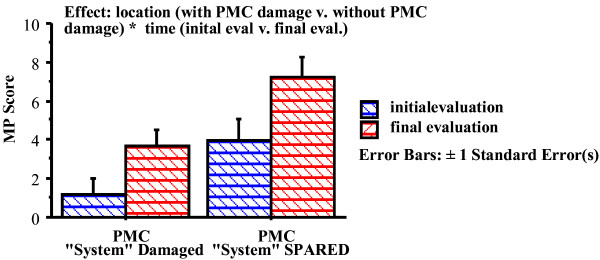
**Outcomes and Pre-Motor Status**.

So far our working model justification attempted to establish temporal relationship between implicit motor learning in unimpaired young subjects with lesion foci, between early implicit motor learning with early recovery, and between late implicit motor learning and late recovery. Miyai has demonstrated that patients with large strokes on the middle cerebral artery territories can have quite distinct outcomes depending on whether the pre-motor region was spared or not [[43] and [44]]. This clinical observation of outcomes might offer further support for our working model. Indeed, when (1) examining a group of sub-acute patients with lesions in the motor execution area who participated in our second robotic rehabilitation study and (2) segregating patients with middle cerebral artery lesions (MCA) involving the pre-motor area (PMC) from those with a spared pre-motor cortex, we observe that patients with spared pre-motor cortex have a better prognosis, supporting the role of the PMC in recovery. Table 2 and figure 1 show the motor power scores of 33 of our sub-acute patients enrolled in our initial studies (14 patients with lesion involving the PMC and 19 patients with spared PMC; see 42). Patients with lesions of comparable volume had different functional outcome depending on whether the PMC was damaged [43]. Results from other investigators using a variety of functional cerebral imaging techniques have also pointed to the PMC as a crucial region of activation during motor recovery [45-47].

**Table 2:** Motor Power Scores at Admission and Discharge of Patients with MCA lesion including or excluding the Pre-Motor Territories

(out of 20)	PMC (14 patients)	SPMC (19 patients)
MP-admission	1.19 ± 0.83	3.95 ± 1.10

MP-discharge	3.66 ± 0.86	7.24 ± 1.02

PMC or sPMC respect; ANOVA for groups being different: p-value 0.0027.

It is also important to emphasize again the impact of the intensity and task-specificity of robotic rehabilitation on recovery [2]. For this second 56 sub-acute patient replication study, a histogram of the number of patients per lesion volume (bins of 25 cm^3^) suggested a bimodal distribution, indicating two distinct classes of patients: one with lesion volumes smaller than 100 cm^3 ^(N = 42) and another with lesions larger than 100 cm^3 ^(N = 14). While an analysis of whether the differences in motor outcome might result from lesion volume alone was unrevealing, of those in the group of 42 patients with smaller lesion volume, who were exposed to an additional 1-hour of high-intensity, task specific robotic sensory-motor training outranked those not exposed to this kind of focused exercise [42].

**Figure 2:**
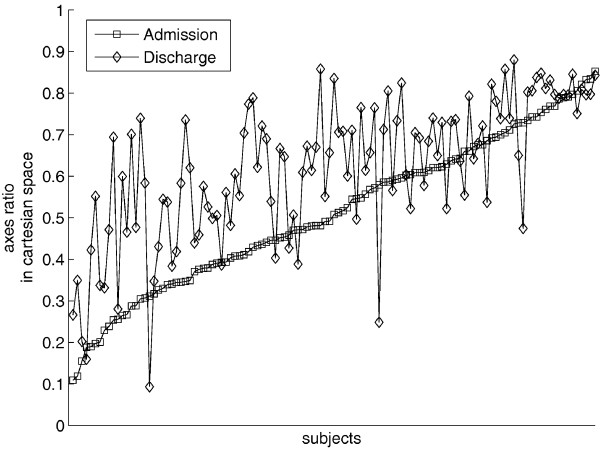
**Circle Drawing**. One-hundred and seventeen (117) persons with chronic impairment due to stroke attempted to draw circles during unassisted evaluation at admission and discharge from 18 robotic sessions. Circle drawing was not part of the training during therapy, which included 1,024 point-to-point movements per therapy session. Difference between admission and discharge is significant (p ≤ 0.05).

We will conclude briefly discussing our selection of a motor learning model versus a motor adaptation model. Dipietro et al examined in persons with chronic impairment due to stroke whether untrained movements were also characterized by changes similar to trained movements [8]. We enrolled persons with chronic impairments following stroke in an 18-session robot-assisted therapy program where subjects trained in point-to-point reaching movements which evoked significant improvements (as measured on clinical and robot scales) by discharge. At the beginning and end of therapy, we asked subjects to perform circle drawing movements, a task for which they had received no training. If these untrained movements displayed changes similar to trained movements, this would provide further insights on movement synergies and coordination, generalization, and support for the theory that Central Nervous System (CNS) generates behavior by combining submovements [8,9,21,22,29,48]. For our purpose here, it would also indicate that a motor learning and not a motor adaptation model is more appropriate as the limb motor control became more exacting for an untrained task and that motor recovery includes features similar to skill learning. Figure 2 shows changes in axis ratio of the ellipse fitted to chronic stroke outpatients' attempts to draw circles. This axis ratio is a metric that indicates the ability of subjects to coordinate inter-limb joint movement (see 8 for more details on this metric). However, as mentioned earlier, we only trained subjects for point-to-point movements, not for circle drawing. This finding extends our understanding of generalization which occurs for the same workspace and limb segment and demonstrates skill learning.

The results above should be viewed with appropriate caution, but they support an emerging understanding of motor recovery that provides hope to improve patient outcomes.

## Conclusion

Experience with over 400 stroke patients has suggested a working model of recovery similar to implicit motor learning. Most strokes preserve the patient's understanding of task goals, but leave an inability to perform the task – even simple tasks. As with implicit learning, recovery occurs without awareness of the learned information. Hence therapy might be more successful if it attempted to inform patients of their progress toward their goals and de-emphasized explicit explanations of the set of muscles or muscle groups that must be activated. While the results presented here are serendipitous in nature, we are testing in very severe to moderate strokes whether motor recovery indeed involves similar brain structures as in implicit motor learning by unimpaired subjects. Ultimately, we plan to apply this knowledge to the design of training paradigms to complement pharmaceutical agents and electrophysiological stimulation that enhance implicit motor learning, potentially opening new routes for greater rehabilitation success.

## Competing interests

H.I.K and N.H are co-inventors in MIT-held patents for the robotic devices used to treat patients in this work. They hold equity positions in Interactive Motion Technologies, Inc., the company that manufactures this type of technology under license to MIT.

## Authors' contributions

This manuscript was drafted by HIK with editorial assistance of BV and NH and it represents shared views amassed during 15 years of close collaboration.
